# The Physician’s Prayer

**Published:** 1914-12

**Authors:** Josephine Daniel


### EDITORIAL DEPARTMENT.

MRS. F. E. DANIEL, Publisher and Managing Editor.
DR. R. H. L. BIBB, Associate Editor.

CONTRIBUTING EDITORS:

Dr. H. Sheridan Baketel, New York, editor *Medical Times:* Dr. M. H. Boerner, Austin, Texas; Dr. G. Henri Bogart, Paris, Ill.; Dr. M. M. Carrick, Dallas, Texas; Dr. Edward H. Carey, Dean Medical Department, Baylor University, Dallas, Texas; Dr. W. L. Crosthwaite, Waco, Texas; Dr. T. D. Crothers, Hartford, Conn.; Dr. H. W. Cummings, Hearne, Texas; Dr. Oscar Dowling, New Orleans, President Louisiana State Board of Health; Havelock Ellis, M. D., London, Eng.; Dr. May Agnes Hopkins, Dallas, Texas; Dr. A. W. Fly, Galveston, Texas; Dr. G. B. Foscue, Waco, Texas; Dr. E. S. Goodhue, Holualoa, Kona, Hawaii; Dr. M. B. Grace, Seguin, Texas; Dr. Joe Gilbert, Austin, Texas; Dr. Charles W. Hughes, St. Louis, editor *Alienist and Neurology*; Dr. James G. Kiernan, Chicago; Dr. George H. Lee, Galveston, Texas; Dr. John T. Moore, Houston, Texas; Dr. Frank Paschal, San Antonio, Texas; Dr. John Preston, Austin, Texas, Superintendent State Lunatic Asylum; Dr. G. Wilse Robinson, Kansas City, Mo.; Dr. Bacon Saunders, Fort Worth, Texas; Dr. Allen J. Smith, Philadelphia, Medical Department, University of Pennsylvania; Dr. Z. T. Scott, Austin, Texas; Dr. Ralph Steiner, Austin, Texas, President Texas State Board of Health; Dr. John S. Turner, Dallas, Texas; Dr. Charles Venable, San Antonio, Texas.

### The Physician's Prayer.

Oh God, thou loving Father of mankind, today with face upturned to Thee I bare my soul—I pray that Thou wilt enable me to lead my life and practice my art in uprightness and honor; help me to realize that mine is a Holy calling and may my service be such that I shall have no shame nor fear in rendering to Thee an account of my stewardship. I pray that Thou wilt so illumine my soul with Thine own effulgence that none of the darkness of impure thoughts, of petty jealousies, bickerings or strife may find a lodgment there.

Grant unto me such a living realization of Thy Great Fatherhood that I shall see in every human being a child of Thine, whom Thou dost love most tenderly. Deliver me from the temptations that daily beset my way, and help me to be loyal to my profession and just and generous to its members.

And when the last call has been made, the last fluttering pulse tested, the last heart beat counted, the last sad heart comforted, as I stand waiting the summons to eternity, I pray that I may have the consciousness that I have wronged no child of Thine, that I have done faithfully and well my share of the world's work—that I have helped to raise a higher standard of ideals and have made smoother the path for those who follow me.

JOSEPHINE DANIEL.

